# Correlates of unequal access to preventive care in China: a multilevel analysis of national data from the 2011 China Health and Nutrition Survey

**DOI:** 10.1186/s12913-016-1426-2

**Published:** 2016-05-12

**Authors:** Chi Huang, Chao-Jie Liu, Xiong-Fei Pan, Xiang Liu, Ning-Xiu Li

**Affiliations:** West China School of Public Health, Sichuan University, Chengdu, China; Chengdu Xindu District Center for Disease Prevention and Control, Chengdu, China; School of Health Management, Hubei University of Chinese Medicine, Wuhan, China; School of Psychology and Public Health, La Trobe University, Melbourne, Australia; School of Public Health, Tongji Medical College, Huazhong University of Science and Technology, Wuhan, China

**Keywords:** Preventive care, General physical examination, Correlates, Equality, Basic social medical insurance, China

## Abstract

**Background:**

Preventive care has an essential role in reducing income-related health inequalities. Despite a general consensus of the need of shifting focus from disease treatment to wellness and prevention, little is known about inequalities in access to preventive care in China. Our study aimed to explore the inequalities in preventive care usage and factors that were associated with such inequalities among Chinese adults.

**Methods:**

Multilevel logistic regression analyses were performed using national data from the 2011 Chinese Health and Nutrition Survey. The study sample comprised 13,483 adults who were covered by Basic Social Medical Insurance (BSMI). We analyzed individual socioeconomic status (marital status, education attainment, annual household income per capita, and medical insurance) and contextual factors for their influence on preventive care usage (region of residence and type of community) after controlling for health needs (age, sex, and health condition).

**Results:**

Out of the participants, 6.9 % received preventive care services over the past four weeks and 3.9 % went for a general physical examination prior to the survey. We noted regional disparities in the overall use of preventive care and specific use of general physical examination, with residents from central and northeastern regions less likely to use preventive care including general physical examination than in the more affluent eastern region. Lower levels of education and income were associated with reduced use of preventive care. Subscriptions to less generous social medical insurance programs such as Urban Resident-based Medical Insurance Scheme or New Rural Cooperative Medical Scheme were associated with decreased specific use of general physical examinations, but not overall use of preventive care.

**Conclusions:**

Inequalities in preventive care usage were evident in China, and were associated with health needs and socioeconomic characteristics. Current health insurance arrangements may fail to reduce inequalities relating to preventive care. A fair and more coherent policy across all BSMI schemes is needed.

## Background

Preventive care has an essential role in reducing income-related health inequalities [1]. People with low incomes are more likely to suffer from ill health, but financial barriers often limit their access to necessary medical treatments [2, 3]. High coverage of preventive services can reduce the uneven distribution of burden of diseases between the poor and rich, and even lower the health care expenditures [4]. For example, preventive services such as immunization and cervical cancer screening are effective in identifying and curbing diseases that contribute to the growth of health expenditure [5]. Preventive care therefore has the potential to progressively benefit the poor.

Despite a general consensus of the need of shifting focus from disease treatment to wellness and prevention, our understanding about how people seek preventive care is limited in China. Instead much hope over the past decade has been placed on universal health coverage to increase access to health services, including preventive care, and to improve health. China has amazed the world with its rapid expansion of social health insurance coverage. Nearly 95 % of the population (1.26 billion) has been covered by the Basic Social Medical Insurance (BSMI), with 424 million people enrolled in the urban areas, and 835 million in the rural areas in 2011 [6].

The BSMI system is designed to match the existing multi-tier service infrastructure in China. It consists of three exclusive programs, the Urban Employee-based Basic Medical Insurance (UEBMI), Urban Resident-based Basic Medical Insurance (URBMI) and New Rural Cooperative Medical Scheme (NRCMS). A Chinese citizen is only entitled to one of the three based on the household registration (rural or urban) and employment status (if he/she is an urban resident). The UEBMI is an employment-based insurance program that covers urban employees and retired workers in the formal sector [7]. Self-employed and rural industry workers are not included in the UEBMI, but can be allowed to buy into the program [8]. The URBMI is established as a voluntary scheme for urban residents, mainly including the unemployed, the elderly, children, and college students [9]. Individual premium contributions are matched and subsidized by central and local governments. The NRCMS is a mutual help and risk-pooling health protection scheme for rural residents. Although it is a voluntary social health insurance program [10], local and central governments contribute more than 75 % of the funds [11]. The majority of URBMI and NRCMS cover inpatient expenditure, although an increasing number of them are expanding to including catastrophic spending on outpatient care [12, 13]. The UEBMI includes a social pooling account for inpatient care and an individual medical savings account (MSA) for outpatient care. The MSA is a private account that allows use in a wider range of settings and services, which may involve preventive care. The BSMI system in China diversifies access to health services for people with different BSMI subscriptions. Rural consumers are encouraged (through higher rates of reimbursement) to maintain loyalty to their township and county facilities, while urban residents are exposed to much more concentrated facilities and high-tech services [14]. National statistics showed that 8.55 health workers per thousand within the population are available for urban residents, compared with 3.41 for rural residents in 2012 [15]. In addition, funds available to people enrolled in different BSMI are vastly different. UEBMI enrollees enjoy an average of 1771 Chinese Renminbi (RMB) (about US$270) per capita annually, compared with 253 RMB for NRCMS enrollees and 219 RMB for URBMI enrollees in 2012 [13].

Studies about health inequalities under the BSMI have depicted a mixed picture in China. On the one hand, health achievements over the past decades have been extraordinary. For example, maternal mortality ratio declined from 85.8 per 100,000 live births in 1996 to 49.3 per 100,000 in 2005-2006 [16]. Much of the progress was due to the implementation of the maternal and child care program aiming at improving preventive care and hospitalized delivery. On the other hand, despite rapid economic growth and expansion of BSMI coverage in China, increased wealth gap and multi-tier BSMI entitlements have caused concerns about equalities in health services and outcomes beyond selective programs [17–19]. Although BSMI has changed the funding structure for health care services, leading to dramatic reduction in the proportion of out-of-pocket payments, inequalities in access to health care has remained a serious issue. While the NRCMS intends to progressively benefit the poor, the two other insurance schemes, especially the URBMI seems to regressively benefit the rich, enlarging the gap in access of health services between rich and poor [20]. Disparities in medical insurance entitlements make a significant contribution to inequalities in health services for those with chronic conditions. NRCMS enrollees experienced similar levels of catastrophic health expenditure and medical impoverishment as those without health insurance [21]. The role of BSMI in reducing health inequality is very limited [22].

Although extensive studies have investigated into inequalities in access to disease treatments [23, 24], little is known about such inequalities in preventive care in China. In many developed countries where high health coverage is achieved, underuse of preventive care is common even when services are free [25]. Some researchers argue that the recent health reform in China has biased towards disease treatments and overlooked preventive care [26]. There is a consensus that a balance should be struck between disease treatment and preventive care in the health system development [27]. In such context, we conducted the study to examine (1) the usage of preventive care services in China; (2) inequalities in preventive care usage; and (3) individual and contextual factors that contributed to the inequalities in preventive care.

## Methods

### Study population

Our analyses were based on data from the 2011 China Health and Nutrition Survey (CHNS) data that was conducted by the National Institute of Nutrition and Food Safety at the Chinese Center for Disease Control and Prevention in collaboration with the Carolina Population Center at the University of North Carolina in Chapel Hill [28, 29]. The 2011 CHNS used a multistage random cluster process to select households and individuals to participate in the survey. Briefly, primary sampling units included nine provinces and three autonomous mega-cities in western (Chongqing, Guangxi and Guizhou), central (Henan, Hubei and Hunan), north-eastern (Liaoning and Heilongjiang), and eastern (Beijing, Shanghai, Jiangsu and Shandong) regions of China. Participating provinces and autonomous mega-cities are diverse in terms of their geographic, demographic, and economic variations, and constituted 47 % of China’s population in 2010. Secondary sampling units included 288 communities comprising 60 urban neighborhoods, 60 suburban neighborhoods, 42 towns, and 126 villages. For secondary sampling, two cities (one large and one small, usually the provincial capital and a lower income city) and four counties (stratified by income, one high-, one low- and two middle-income) were selected from each selected province/mega-city. Two urban and two suburban communities from each selected lower income city, one community from the province capital city, and three rural villages from each selected county were randomly selected. Twenty households were randomly chosen from each community/village, and all household members were interviewed.

The survey collected data through face-to-face interviews and blood sampling including key public health risk factors, and health outcomes, demographic, social and economic factors in depth at the individual, household and community levels. Our analyses focused on data of individual demographic, socioeconomic, and geographical characteristics, and preventive care usage immediately prior to the date of survey.

A total of 27,447 individuals participated in the survey in 2011. We excluded children younger than 18 years old because they were financially dependent on their parents. In addition, because few household members were not covered by the BSMI, we only included BSMI (UEBMI, URBMI, or NRCMS) enrollees to facilitate statistical analyses. According to such criteria, we had a final sample of 13,483 including 3,472 (25.75 %) UEBMI enrollees, 2,178 (16.15 %) URBMI enrollees and 7,833 (58.10 %) NRCMS enrollees.

The University of North Carolina and the China Center for Disease Control and Prevention reviewed and approved the procedures for the 2011 CHNS data collection. Written informed consent was obtained from individual participants. Our study was approved by the Ethics Committee of West China School of Public Health, Sichuan University.

### Correlates and outcome measures for analysis

#### Demographic and socioeconomic characteristics

Major demographic characteristics were age and sex. Age was categorized into three groups (18 to 44, 45 to 59, and 60 and above) for our analysis, and sex was male or female.

Socioeconomic variables include marital status, education attainment, and household income per capita. Marital status was categorized as married, never married, and others (including divorced, widowed and separated). Education attainment was classified into five levels including no formal education, primary school, middle school, high school (including vocational training), and university. Household income was estimated using a validated instrument consisting of detailed questions on income and expenditure. The household income was regarded as the sum of all nine potential sources of income (business, farming, fishing, gardening, livestock, non-retirement wages, retirement income, subsidies, and other income) and revenue minus expenditures. Annual household income per capita was used in our study. Details of the instrument and algorithms were provided elsewhere [30].

#### Health conditions

Self-reported acute illness and chronic conditions were taken into account and dichotomized. Survey participants were asked whether they experienced any acute illness over the past four weeks, and to rate their experience in a nominal scale: 0 = no illness and 1 = illness. Chronic conditions were defined as a history of diagnosis made by doctors regarding high blood pressure, diabetes, myocardial infarction, transient ischemic attack, stroke, cancer, asthma, or chronic obstructive pulmonary disease.

#### Types of medical insurance

Survey participants were classified based on their subscription to any of the three mutually exclusive types of BSMI, i.e., UEBMI, URBMI, and NRCMS. In addition, they were asked whether they purchased any type of commercial medical insurance.

#### Residency characteristics

Residency characteristics (i.e., contextual factors) included region of residence and type of community. They were grouped according to socioeconomic development and geographic distribution of secondary sampling units into western, central, eastern, and northeastern China, and urban neighborhood, suburban village (neighborhood), county town neighborhood, and village.

#### Preventive care usage

We used two variables that measure preventive care usage over the past four weeks prior to the survey: overall use of preventive care and specific use of general physical examinations. The two variables were estimated based on two questions about whether the participant received any preventive care services and what type of preventive care services the participant received during the past four weeks. Both variables were dichotomized (yes/no) according to responses from the participants. Preventive care services include general physical examinations, and selective programs designed for detecting particular diseases in certain target populations (such as prenatal examination, postnatal examination, gynecological examination, blood pressure screening, cancer screening, and vision or hearing examination). The selective programs are often pre-packaged (such as maternal care, child care, and geriatric care) and promoted by public health agencies. Some selective programs may be fully funded by governmental budgets, while others may be treated as an integral part of disease management regimens and covered by health insurance schemes. General physical examinations are usually consumer-driven and are not specific to a particular disease. It varies significantly in scope and content, and is usually paid out of pocket or by the employer.

#### Statistical analysis

Preventive care usage might be associated with both needs and non-needs factors [31]. In our analyses, demographic and socioeconomic characteristics, health condition, and medical insurance were regarded as needs variables, while residency (region and type of community) and medical insurance were non-needs variables.

We summarized needs and non-needs variables, and the overall use of preventive care and specific use of general physical examinations by percentage, and tested variability in preventive care usage among participants with different characteristics by using chi-square tests.

We developed four regression models stepwise to identify correlates with overall use of preventive care and specific use of general physical examinations. We used multilevel logistic modeling since the sampling frame involved five levels: province/mega-city, county/city, neighborhood, household, and individual. Firstly, we constructed an empty model (Model A) with only a random intercept that allowed us to determine possible contextual dimensions to be included in further modelling. We found that only variances at the household and neighborhood levels showed statistical significance. Thus we applied a three-level logistic regression analysis. Secondly, we included individual characteristics (age, sex, self-reported illness, marital status, education attainment, annual household income per capita, and medical insurance) in the first model. The second model (Model B) investigated the extent to which household differences nested in neighborhood were explained by the individual composition. Thirdly, we included contextual characteristics (i.e., region of residence and type of community) in the first model. The third model (Model C) indicated to which extent regional and urban-rural differences contributed to the differences in preventive care usage. Finally, all individual and contextual variables that showed statistical significance in the second and third models were included in the fourth model (Model D).

Odds ratios (ORs) with 95 % confidence intervals (CIs) were calculated for variables in different models. Statistical analyses were conducted using IBM SPSS 21 (Armonk, New York, USA). All tests were two-tailed with a significance level of 5 %.

## Results

### Characteristics of study participants

Participants had a mean age of 51.9 (standard deviation: 15.2) years. 83.8 % were married, and 59.6 % completed middle school or higher education. The median annual household income per capita reached 14,423 RMB (about US$2230). 17.8 % experienced acute illness over the four weeks prior to the survey, while 19.9 % suffered from chronic conditions (Table 1).

**Table 1:** Characteristics of study participants

Characteristics of participants	Frequency	Percentage (%)
Demographic Characteristics		
Age		
18-44	4489	33.3
45-59	4821	35.7
60+	4173	31.0
Sex		
Female	7043	52.2
Male	6440	47.8
Socioeconomic status		
Marital status		
Never married	743	5.5
Married	11292	83.8
Other	1448	10.7
Educational attainment		
No formal education	3073	22.8
Primary school	2376	17.6
Middle school	4192	31.1
High school	2517	18.7
University	1325	9.8
Annual household income per capita (RMB)*		
< 4370 (≤20 percentile) ~ (poorest 20 percentage)	2697	20.0
4370-8434 (≤40 percentile)	2696	20.0
8435-13540 (≤60 percentile)	2698	20.0
13541-21107 (≤80 percentile)	2693	20.0
> 21107 (>80 percentile)	2699	20.0
Health condition		
Illness over past 4 weeks		
No	11083	82.2
Yes	2400	17.8
Chronic conditions		
No	10806	80.2
Yes	2677	19.9
Medical insurance		
CMI		
Yes	421	3.1
No	13062	96.9
BSMI		
NRCMS	7833	58.1
BMIUR	2178	16.2
BMIUE	3472	25.8
Residency		
Region of residence		
Northeastern	1904	14.1
Western	3528	26.2
Central	3619	26.8
Eastern	4432	32.9
Type of community		
Urban neighborhood	2121	15.7
Suburban village	2738	20.3
County town	1822	13.5
Rural village	6802	50.5

Note: ^*^1RMB = US$0.15; CMI: Commercial Medical Insurance; BSMI: Basic Social Medical Insurance; UEBMI: Urban Employees Basic Medical Insurance; URBMI: Urban Residents Basic Medical Insurance; NRCMS: New Rural Cooperative Medical Scheme

### Use of preventive care among participants with different characteristics

Nine hundred twenty nine (6.9 %) visited a doctor for preventive care services, and, in particular, 525 (3.9 %) received a general physical examination in the past four weeks prior to the survey. Older people (8.7 %), people with higher income (≧80 % percentile, 10.0 %), and those who had received university education (11.4 %), those who suffered illness over the past four weeks (14.6 %) or chronic conditions (13.4 %), who enrolled in a commercial insurance program (10.0 %) or the UEBMI (10.1 %), and who resided in a better-off region (eastern, 10.5 %) and neighborhood (urban, 9.7 %) seemed to use preventive care as well as general physical examinations more (Table 2). Never-married participants were less likely to use preventive care services than their married (current or past) counterparts, but not the specific use of general physical examinations.

**Table 2:** Overall use of preventive care and specific use of general physical examinations in study participants

Characteristics of participants	Overall use of preventive care			General physical examinations		
	No.	%	*P*	No.	%	*p*
Demographic Characteristics						
Age						
18-44	253	5.6	<0.001	160	3.6	0.002
45-59	315	6.5		166	3.4	
60+	361	8.7		199	4.8	
Sex						
Female	511	7.3	0.080	264	3.8	0.361
Male	418	6.5		261	4.1	
Socioeconomic status						
Marital status			0.003			0.736
Never married	36	4.9		26	3.5	
Married	768	6.8		446	4.0	
Other	125	8.6		53	3.7	
Education attainment			<0.001			<0.001
No formal education	189	6.2		90	2.9	
Primary school	128	5.4		74	3.1	
Middle school	273	6.5		133	3.2	
High school	188	7.5		116	4.6	
University	151	11.4		112	8.5	
Per capita household income			<0.001			<0.001
≤ 20 percentile	142	5.3		60	2.2	
≤ 40 percentile	122	4.5		72	2.7	
≤ 60 percentile	167	6.2		92	3.4	
≤ 80 percentile	227	8.4		127	4.7	
> 80 percentile	271	10.0		174	6.5	
Health condition						
Illness over past 4 weeks						
No	578	5.2	<0.001	355	3.2	<0.001
Yes	351	14.6		170	7.1	
Chronic conditions						
No	570	5.3	<0.001	363	3.4	<0.001
Yes	359	13.4		162	6.1	
Medical insurance						
CMI			0.011			
Yes	42	10.0		26	6.2	0.014
No	887	6.8		499	3.9	
BSMI			<0.001			
NCRMS	393	5.0		203	2.6	<0.001
URBMI	184	8.5		101	4.6	
UEBMI	352	10.1		221	6.4	
Residency						
Region of residence			<0.001			<0.001
Northeastern	65	3.4		43	2.3	
Western	196	5.6		95	2.7	
Central	205	5.7		127	3.5	
Eastern	463	10.5		260	5.9	
Type of community			<0.001			<0.001
Urban neighborhood	206	9.7		109	5.1	
Suburban village	220	8.0		131	4.8	
County town	133	7.3		88	4.8	
Rural village	370	5.4		197	2.9	

*CMI* Commercial Medical Insurance, *BSMI* Basic Social Medical Insurance, *UEBMI* Urban Employees Basic Medical Insurance, *URBMI* Urban Residents Basic Medical Insurance, *NRCMS* New Rural Cooperative Medical Scheme

### Correlates of overall use of preventive care and specific use of general physical examinations

Several factors at individual, household and community levels were correlated with overall use of preventive care usage (Table 3) and specific use of general physical examinations (Table 4).

**Table 3:** Multilevel regression coefficients and OR estimates for overall use of preventive care

Variables	Model B	Model C	Model D
	OR (95 % CI)	OR (95 % CI)	OR (95 % CI)
Demographic characteristics			
Age			
18–44	0.71 (0.55–0.91)		0.70 (0.54–0.91)
45–59	0.82 (0.67–1.02)		0.82 (0.67–1.02)
60+	Reference		Reference
Sex			
Female	1.19 (1.02–1.39)		1.18 (1.00–1.38)
Male	Reference		Reference
Socioeconomic status			
Marital status			
Married	1.24 (0.83–1.85)		1.24 (0.83–1.86)
Other	1.54 (0.97–2.45)		1.53 (0.96–2.45)
Never married	Reference		Reference
Education attainment			
No formal education	0.52 (0.36–0.76)		0.54 (0.37–0.78)
Primary school	0.57 (0.38–0.80)		0.59 (0.41–0.84)
Middle school	0.70 (0.53–0.94)		0.72 (0.54–0.97)
High school	0.70 (0.53–0.93)		0.71 (0.53–0.93)
University	Reference		Reference
Annual household income per capita			
≤ 20 percentile	0.67 (0.49–0.90)		0.68 (0.50–0.93)
≤ 40 percentile	0.59 (0.43–0.79)		0.60 (0.44–0.81)
≤ 60 percentile	0.74 (0.57–0.97)		0.75 (0.57–0.98)
≤ 80 percentile	0.89 (0.70–1.12)		0.88 (0.70–1.12)
> 80 percentile	Reference		Reference
Health condition			
Illness over past 4 weeks			
Yes	2.36 (1.97–2.83)		2.29 (1.91–2.75)
No	Reference		Reference
Chronic condition			
Yes	1.95 (1.62–2.35)		1.93 (1.60–2.33)
No	Reference		Reference
Medical insurance			
CMI			
Yes	1.21 (0.80–1.82)		1.15 (0.76–1.73)
No	Reference		Reference
BSMI			
NCRMS	0.68 (0.51–0.89)		0.77 (0.57–1.05)
URBMI	0.81 (0.64–1.04)		0.81 (0.63–1.03)
UEBMI	Reference		Reference
Residency			
Region of residence			
Northeastern		0.31 (0.19–0.51)	0.37 (0.23–0.61)
Western		0.54 (0.37–0.80)	0.74 (0.50–1.10)
Central		0.45 (0.30–0.66)	0.60 (0.41–0.90)
Eastern		Reference	Reference
Type of community			
Urban neighborhood		2.26 (1.52–3.35)	1.261 (0.80–1.99)
Suburban village		1.68 (1.13–2.50)	1.21 (0.80–1.84)
County town		1.45 (0.91–2.30)	1.14 (0.70–1.85)
Rural village		Reference	Reference
Random parameters	Coefficient (SE)	Coefficient (SE)	Coefficient (SE)
Level 3: community	0.97 (0.000)	1.02 (0.000)	0.96 (0.000)
Level 2: household	0.94 (0.000)	0.97 (0.000)	0.95 (0.000)

*CMI* Commercial Medical Insurance, *BSMI* Basic Social Medical Insurance, *UEBMI* Urban Employees Basic Medical Insurance, *URBMI* Urban Residents Basic Medical Insurance, *NRCMS* New Rural Cooperative Medical Scheme, *OR* Odds ratio, *CI* Confidence interval, *SE* Standard error

**Table 4:** Multilevel regression coefficients and OR estimates for specific use of general physical examinations

Parameters	Model B	Model C	Model D
	OR (95 % CI)	OR (95 % CI)	OR (95 % CI)
Demographic characteristics			
Age			
18–44	0.59 (0.43–0.82)		0.59 (0.42–0.82)
45–59	0.70 (0.53–0.93)		0.70 (0.53–0.93)
60+	Reference		Reference
Sex			
Female	0.99 (0.81–1.22)		0.99 (0.81–1.21)
Male	Reference		Reference
Socioeconomic status			
Marital status			
Married	1.10 (0.68–1.77)		1.09 (0.68–1.77)
Other	1.02 (0.57–1.84)		1.11 (0.56–1.84)
Never married	Reference		Reference
Education attainment			
No formal education	0.43 (0.27–0.69)		0.45 (0.28–0.71)
Primary school	0.49 (0.32–0.76)		0.51 (0.33–0.80)
Middle school	0.44 (0.31–0.64)		0.45 (0.32–0.65)
High school	0.61 (0.44–0.84)		0.61 (0.44–0.85)
University	Reference		Reference
Annual household income per capita			
≤ 20 percentile	0.51 (0.34–0.78)		0.53 (0.35–0.81)
≤ 40 percentile	0.65 (0.44–0.94)		0.66 (0.45–0.97)
≤ 60 percentile	0.75 (0.53–1.05)		0.75 (0.53–1.07)
≤ 80 percentile	0.85 (0.64–1.15)		0.85 (0.63–1.15)
> 80 percentile	Reference		Reference
Health condition			
Illness over past 4 weeks			
Yes	2.03 (1.59–2.59)		1.98 (1.55–2.53)
No	Reference		Reference
Chronic condition			
Yes	1.28 (0.99–1.65)		1.26 (0.98–1.63)
No	Reference		Reference
Medical insurance			
CMI			
Yes	1.29 (0.78–2.13)		1.23 (0.74–2.03)
No	Reference		Reference
BSMI			
NCRMS	0.57 (0.40–0.82)		0.65 (0.43–0.97)
URBMI	0.72 (0.53–0.99)		0.72 (0.53–0.98)
UEBMI	Reference		Reference
Residency			
Region of residence			
Northeastern		0.38 (0.21–0.69)	0.48 (0.25–0.812)
Western		0.45 (0.28–0.73)	0.65 (0.40–1.07)
Central		0.42 (0.26–0.69)	0.61 (0.37–1.00)
Eastern		Reference	Reference
Type of community			
Urban neighborhood		2.32 (1.42–3.80)	1.10 (0.62–1.95)
Suburban village		1.87 (1.14–3.06)	1.20 (0.71–2.02)
County town		1.68 (0.95–2.97)	1.14 (0.62–2.11)
Rural village		Reference	Reference
Random parameters	Coefficient (SE)	Coefficient (SE)	Coefficient (SE)
Level 3: community	1.26 (0.000)	1.37 (0.000)	1.29 (0.000)
Level 2: household	1.04 (0.000)	1.08 (0.000)	1.06 (0.000)

*CMI* Commercial Medical Insurance, *BSMI* Basic Social Medical Insurance, *UEBMI* Urban Employees Basic Medical Insurance, *URBMI* Urban Residents Basic Medical Insurance, *NRCMS* New Rural Cooperative Medical Scheme, *OR* Odds ratio, *CI* Confidence interval, *SE* Standard error

#### Overall use of preventive care

Participants aged 18-44 years were 0.70 times as likely to use preventive care services as those aged 60 or over (Table 3). Compared with men, women were 1.18 (95 % CI: 1.00-1.38) times more likely to use preventive care services. Participants with ill health conditions (either acute illness or chronic conditions) were about twice as likely to use preventive care as their healthier counterparts. Compared with university-educated participants, less educated participants were less likely to use preventive care services. All participants with an income below the 60 % range were less likely to use preventive care services than those within the top 20 % income range. There was no statistically significant difference between NCRMS and URBMI enrollees and UEBMI enrollees. Participants living in central and northeastern regions were 0.60 and 0.37 times as likely to use preventive care services as those living in better-off eastern region.

#### Specific use of general physical examinations

Similar to the overall use of preventive care, younger participants (18–44 and 45–59 years old) were 0.59 and 0.70 times as likely to take a general physical examination as those aged 60 or older. Participants with acute illness were 1.98 times more likely to have general physical examinations compared with their healthier counterparts. Less educated participants were less likely to take general physical examinations than those with a university level education. Participants with an income below the 40 percentile were 0.53 and 0.66 times as likely to have general physical examinations as those among the top 20 % range of income. NCRMS and URBMI enrollees were 0.65 and 0.72 less likely to take general physical examinations as UEBMI members. Significant gaps in the use of general physical examinations was found between northwestern (OR = 0.48) and central (OR = 0.61) regions, and eastern region.

## Discussion

Our study found that 6.9 % of study participants used preventive care services, and in particular, 3.9 % received a general physical examination. However, our estimates were based on self-report for the last four weeks, and those who did not report use of preventive care may have reported receiving this care had the recall period been extended to longer time, for example, one year. In contrast, estimates based on the 2006 CHNS data showed that only 2.8 % Chinese adults used preventive care services and 1.2 % used a general physical examination in the past four weeks [32]. In this regard, preventive care usage in China had tripled in five years.

Older age, female, and health problems were associated with higher preventive care usage in our study, which is consistent with the proposition that health needs factors play an important role in service utilization [33, 34]. However, sex and chronic condition did not impact the specific use of a general physical examination. This is possibly because the study participants preferred a more selective approach in physical examinations and those with the largest need (such as women and those living with chronic conditions) may receive physical examinations under free public health service packages or during medical procedures [35]. A total of 41 packages are defined by the Chinese government as essential public health services under ten categories, and are delivered free of charge through public health agencies (including local centers for disease control and prevention and primary care facilities). Most of these services are selective, targeting a specific group of population such as women (eg. postnatal home visits and breast cancer screening), children (eg. physical development monitoring and vaccinations), elderly (eg. diabetes screening), and people with chronic conditions (eg. management of hypertension, type II diabetes, and severe mental illness) [36]. Of note, the high overall uptake of preventive care among people with health problems, particularly chronic conditions might also be explained by the fact that some preventive services are disease-related and were thus used for the monitoring of disease progression. Although there is debate regarding the benefits and harms of general physical examinations among the symptomless [37, 38], these are generally considered as important measures to prevent diseases in China, particularly among vulnerable groups such as the old, the poor, and those in rural areas. However, general physical examinations are usually requested by consumers (not by public health agencies) in China, and are mostly not covered by public health service packages. Consumers are required to pay for such services, leading to universally low uptake of general physical examinations, and no evident variability between males and females, and between people with and without chronic conditions.

Our study showed that disparities in usage of preventive care and general physical examinations were attributable to the socioeconomic status. Education attainment and annual household income per capita were positively associated with preventive care utilization. This finding is consistent with results of studies undertaken elsewhere [39–41]. Understandably, a higher level of awareness of the needs of preventive care is associated with university education [25, 42]. Some preventive care services such as cancer screening may impose fear and anxiety on consumers. This is a great challenge, especially for those who have a low level of formal education [43]. Financial burden may present a significant barrier for the uptake of preventive care due to lower discretionary buying power of the poor. Empirical evidence shows that basic living needs like food and housing consumes a large proportion of disposable income of the poor in China [44]. Many poor people would forsake much needed medical care [45] as well as preventive care services, when they have to sacrifice their basic living budget for these services. This resonates with the inverse care law that “the availability of good medical care tends to vary inversely with the need for it” [46]. Since the reimbursements from BSMI are generally low in China, health care is largely exposed to market forces and thus the less well-off tend to use preventive care less.

NCRMS and URBMI enrollees were less likely to register for general physical examinations than UEBMI enrollees. Although the Chinese BSMI has not been designed for promoting preventive care alone, unintended consequence of negative discrimination against the poor in relation to preventive care is obviously undesirable. UEBMI enrollees have enjoyed better access to preventive care than NCRMS and URBMI enrollees. In addition, URBMI and NRCMS members do not have a MSA that can be drawn for paying for preventive care services. The existence of multi-tier health insurance entitlements by itself is an indication of inequality. It is unlikely that the current health insurance arrangements in China could play a significant role in reducing inequality in preventive care usage.

Our study found that residents living in a central or northeastern region were less likely to uptake preventive care compared with residents living in an affluent eastern region. Large regional disparities in health and health care services were also noted in other studies undertaken in China [47, 48]. China has developed a devolved fiscal responsibility system in health care. The affluent eastern regional governments have a greater capacity to invest in health for their constituents compared with their less affluent counterparts [49, 50]. For example, BSMI enrollees from the eastern region are generally entitled to a higher level of benefits compared with others, in terms of deduction and co-payment requirements, scope of services covered, and flexibility of using funds to pay for preventive care services [9, 51]. Since regional disparities in preventive care usage remain a serious issue, heavy subsidies from the central government to the poorest western region may still be needed to diminish the gap in preventive care usage between wealthy and poor region.

We were surprised to find that urban-rural disparities in preventive care usage were not statistically significant in our study: this sharply contrasts with large urban-rural gaps in medical treatment services identified in previous studies [52]. The absence of urban-rural disparity in preventive care may, however, simply be a reflection of overall low level of preventive care usage in both rural and urban China. Closing urban-rural gap in health services has been one of the core policy goals of the recent health reform in China [53]. Considering existing urban-rural disparities in socioeconomic development and burden of disease, a positive discrimination (favorable policies towards the poor) in preventive care towards rural populations is preferable.

The findings of this study have significant policy implications. Some researchers have doubted the capacity of health insurance policies in preventing or at least alleviating medical impoverishment in China [54]. Inadequate funds in BSMI have struggled to meet the needs of rapid escalation of medical bills [55, 56]. Arguably, if BSMI can increase preventive care usage, we may reasonably expect fewer catastrophic financial imposts arising from medical events. This study indicates a better health insurance plan (such as the UEBMI with MSA) may have the potential to encourage uptake of preventive care services. As proposed by the theory of fundamental causes to health disparities [57], minimizing the extent to which individuals can use their socioeconomic resources to achieve a health advantage can help to break the link between socioeconomic resources and preventive care services [58]. In this regard, a more equitable health insurance system is needed to maximize the potential, especially for disadvantaged populations in disadvantaged areas. Efforts such as the consolidation of all three BSMI schemes to narrow disparities in benefit packages ought to be prioritized [20].

This study has a number of limitations to address. Firstly, this study over-represents individuals residing in Eastern China. Any further generalization should be made cautiously. Secondly, measurements in relation to preventive care usage were captured by only a few simple questions. It is unclear what exact services were delivered to the participants. This prevents us from further determination of the scope of services that needs to be extended. Thirdly, the recall period was relatively short. Participants were asked to report use of preventive care over past four weeks prior to the survey. Such a short recall period may help reduce recall bias, but it may not actually capture the actual use of preventive services. Fourthly, our analyses excluded the residents without subscription to any of the three types of BSMI. The uninsured might differ from those enrolled in BSMI in aspects such as health conditions and socioeconomic status. The exclusion might partly influence the generalizability of our results. Fifthly, individuals with specific characteristics (such as the rich) might be more likely to self-report illnesses, so the associations between these characteristics and use of preventive care might be underestimated. Despite these limitations, we believe our findings may still be relevant for the provision of evidence for inequalities in preventive care in China.

## Conclusions

Inequalities in preventive care usage were evident in China, and were associated with both health needs and socioeconomic characteristics. Overall, the level of preventive care usage was low in those who had low income, were without a tertiary education, lived in a less affluent region, and subscribed to a less affluent insurance program scheme (either UEBMI or NRCMS). Current health insurance arrangements may fail to reduce inequalities relating to preventive care. A fair and more coherent policy across all BSMI schemes is needed.

### Ethics approval and consent to participate

The University of North Carolina and the China Center for Disease Control and Prevention reviewed and approved the procedures for the 2011 CHNS data collection. Written informed consent was obtained from individual participants. Our study was approved by the Ethics Committee of West China School of Public Health, Sichuan University.

### Consent for publication

Not applicable.

### Availability of data and materials

Our study was solely based on the 2011 CHNS data, which is publicly available through the University of North Carolina Carolina Population Center.

## Abbreviations

BSMI: Basic Social Medical Insurance
CHNS: China Health and Nutrition Survey
CIs: confidence intervals
MSA: medical savings account
NRCMS: New Rural Cooperative Medical Scheme
ORs: odds ratios
RMB: Renminbi
SE: standard error
UEBMI: Urban Employee-based Basic Medical Insurance
URBMI: Urban Resident-based Basic Medical Insurance
